# On the Mathematical Analysis of Ebola Hemorrhagic Fever: Deathly Infection Disease in West African Countries

**DOI:** 10.1155/2014/261383

**Published:** 2014-09-11

**Authors:** Abdon Atangana, Emile Franc Doungmo Goufo

**Affiliations:** ^1^Institute for Groundwater Studies, Faculty of Natural and Agricultural Sciences, University of the Free State, Bloemfontein 9300, South Africa; ^2^Department of Mathematical Sciences, University of South Africa, Florida Sciences Campus, Florida 0003, South Africa

## Abstract

For a given West African country, we constructed a model describing the spread of the deathly disease called Ebola hemorrhagic fever. The model was first constructed using the classical derivative and then converted to the generalized version using the beta-derivative. We studied in detail the endemic equilibrium points and provided the Eigen values associated using the Jacobian method. We furthered our investigation by solving the model numerically using an iteration method. The simulations were done in terms of time and beta. The study showed that, for small portion of infected individuals, the whole country could die out in a very short period of time in case there is not good prevention.

## 1. Introduction

We shall recall that the filoviruses belong to a virus family named Filoviridae. This virus can cause unembellished hemorrhagic fever including humans and also monkeys [1–3]. In the literature, only two members of this virus family have been distinguished, namely,* Marburgvirus* and* Ebolavirus*. However, so far only in the literature five species of* Ebolavirus* have been identified including: Ivory Coast, Sudan, Zaire, Reston, and Bundibugyo. Amount these five species, Ebola virus is the only member of the Zaire* ebolavirus* species and the most dangerous, being responsible for largest number of outbreaks [1–4]. Ebola is an unusual nevertheless fatal virus that causes bleeding inside and outside the body [4]. As the virus spreads through the body, it damages the immune system and organs. Ultimately, it causes levels of blood-clotting cells to drop [5]. This leads to severe, uncontrollable bleeding [5].

**Figure 1:**
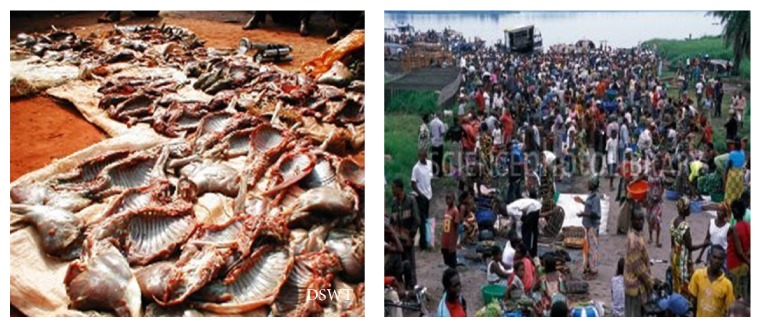
Bush meat market in a Sub-Saharan African country.

**Figure 2:**
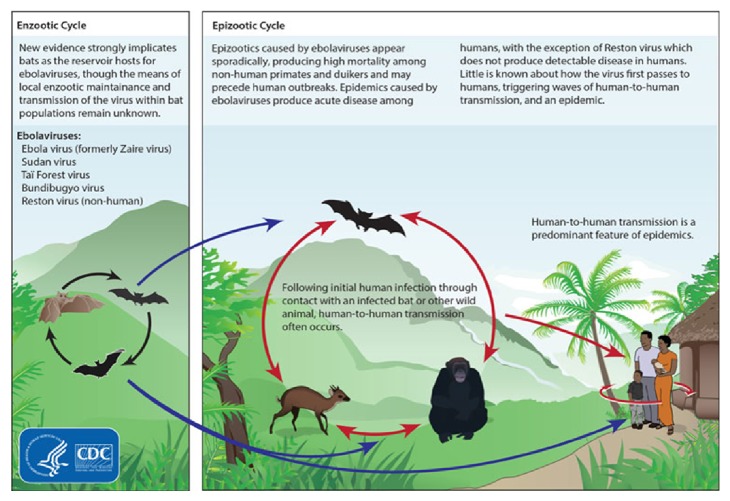
This graphic shows the life cycle of the* ebolavirus*. Bats are strongly implicated as both reservoirs and hosts for the* ebolavirus* (reference: Center of disease control and prevention).

One of the big challenges faced by West African countries is lack of employments. It is estimated that about 133 million young people (more than 50 percent of the youth population) in Africa are illiterate. Many young people have little or no skills and are therefore largely excluded from productive economic and social life. Those that have some education often exhibit skills irrelevant to current demand in the labour market, in a situation where educational and skill requirements are increasing, resulting in millions of unemployed and underemployed youth. The incidence of youth unemployment in Sub-Saharan African is estimated to be over 20 percent. Therefore, to provide for their family daily needs, many families in West and Central Africa reply on farming, fishing, and hunting. In the case of hunt, they will kill many wild animals, sometime dry and supply to the rural markets. Some images underpinning this situation are depicted in Figure 1. These pictures show a trade of these bust meats in some rural area in a Sub-Sahara country.  Figure 2 shows the life cycle of the* ebolavirus* and Table 1 shows parameters used for simulations according to some reported data.

Incidentally, EVD (Ebola Virus Disease) is believed to take place after an Ebola virus is transmitted to an initial human by contact with an infected animal's body fluid. On the other hand, human-to-human transmission can take place with direct contact with blood or bodily fluid from an infected person. Fruit bats are considered to be the most likely natural source of the Ebola virus. As initial transmission, the bat drops incompletely eaten fruits and pulp and then lands animals for instance, gorilla feed fallen fruits, and then the hunter kills and sells the body of the affected animal. Now the affected human is in contact with the rest of his family. The chain of transmission can be viewed in the following picture.

Since all physical problems can be modeled via mathematical equation, we aim in this paper to analyze the spread of this deadly disease using mathematical equations. We shall propose a model underpinning the spread of this disease in a given Sub-Saharan African country in the next section.

## 2. Mathematical Formulation

Let us consider a West African country with a total number of populations *N* at a given time. Let us assume that the rate of death caused by natural death and other diseases is factored out to be *α*. *s*,  *i*,  *r*, and *d* are to be rate of infection by Ebola, rate of recovery, rate of susceptibility, and rate of death by Ebola, respectively. In this work, *S*(*t*),  *I*(*t*),  *R*(*t*), and *D*(*t*) will describe the susceptible, the infected, the recovery, and the total death populations, respectively. Therefore, the mathematical equation describing the rate of change of susceptible population is given as follows:

(1) $$\begin{array}{c} \frac{dS\left(t\right)}{dt}=-iS\left(t\right)I\left(t\right)+sR\left(t\right)-\alpha N. \end{array}$$

The above equation is obtained because *i* is the rate of infectious persons from recovery population turned out to be vulnerable again at the rate *s* and finally the quantity of population that die with natural death and other diseases at the rate *α*. The differential describing the rate of change of infected group is provided as

(2) $$\begin{array}{c} \frac{dI\left(t\right)}{dt}=iS\left(t\right)I\left(t\right)-dI\left(t\right)-rI\left(t\right). \end{array}$$

The above equation can be justified as follows: the total number of persons removed from susceptible group can be mathematically expressed as *iS*(*t*)*I*(*t*). However, due to the introduction of medication, a number of individuals will be recovered at a rate of *r* and also a quantity of infected person will die at a rate *d*. The rate of recovery population is described by the following ordinary differential equation:

(3) $$\begin{array}{c} \frac{dR\left(t\right)}{dt}=rI\left(t\right)-sR\left(t\right). \end{array}$$

The above is quite easy to be obtained. Finally the rate of change of death in that given country will be described via the ordinary equation

(4) $$\begin{array}{c} \frac{dD\left(t\right)}{dt}=dI\left(t\right)+\alpha N. \end{array}$$

Thus, the set of mathematical equations involved can then be given as

(5) dStdt=−iStIt+sRt−αN,dItdt=iStIt−dIt−rIt,dRtdt=rIt−sRt,dDtdt=dIt+αN.

Although the above equation is novel and valid, we have employed the common Newtonian idea of rate of change which has been intensively disparaged to not describe accurately the real rate of change in time [6–10]. Therefore, we shall consider modifying the above equation using a derivative that can take into account the time scale and also obeys the classical properties of the Newtonian derivative, for instance, chain rule and others. This derivative was recently proposed in the work [11] and defined as

(6) D0AxβfxM=lim⁡ε→0⁡fx+εx+1/Γβ1−β−fxε,

for all *x* ≥ *a*, *β* ∈ (0,1]. Then, if the limit of the above exists, *f* is said to be *β*-differentiable.

**Theorem 1 (see [11]).**

Assuming that *f* is differential and *β*-differentiable the on the opened interval (*a*, *b*), then

(7) D0AxβfxM=x+1Γβ1−βlim⁡h→0⁡fx+h−fxh.

**Definition 2 (see [11]).**

Let *f* : [*a*, *∞*) → *R* be given function, and then we propose that the integral of order *β*-integral of *f* is

(8) IaAxβfx=∫axt+1Γββ−1ftdt.

The above operator is the inverse operator of the proposed beta derivative and is called the Atangana-beta integral. Now using the above derivative, we change (5) to

(9) D0AtβSt=−iStIt+sRt−αN,D0AtβIt=iStIt−dIt−rIt,D0AtβRt=rIt−sRt,D0AtβDt=dIt+αN.

The above equation shall be referred to as Atangana's Beta Ebola System of Equations (ABESE). We shall in the next section give a detailed analysis of the ABESE.

## 3. Analysis and Validation

### 3.1. Validation

The first aspect of this analysis is to verify that the _0_^*A*^ *D*_*t*_^*β*^ *S*(*t*) + _0_^*A*^ *D*_*t*_^*β*^ *R*(*t*) + _0_^*A*^ *D*_*t*_^*β*^ *I*(*t*) + _0_^*A*^ *D*_*t*_^*β*^ *D*(*t*) = 0 such that *S*(*t*) + *R*(*t*) + *I*(*t*) + *D*(*t*) = 0. Indeed, by adding equations in system (9), we have

(10) D0AtβSt+D0AtβRt+D0AtβIt+D0AtβDt=0.

Now, using the linearity of the Beta-derivative, we have

(11) D0AtβSt+Rt+It+Dt=0.

Now applying the inverse operator of _0_^*A*^ *D*_*t*_^*β*^ given in Definition 2, we obtain

(12) $$\begin{array}{c} S\left(t\right)+R\left(t\right)+I\left(t\right)+D\left(t\right)=\text{constance}=N. \end{array}$$

### 3.2. Endemic Equilibrium Points

We shall use only the first three equations of system (9) to find the endemic equilibrium point. Thanks to the Beta-derivative that allows us to have that, a beta derivative of a constant is zero because the equilibrium points are obtained here by assuming that the solution of (9) does not depend on the time; then,

(13) 0=−iS∗I∗+sR∗−αN,0=iS∗I∗−dI∗−rI∗,0=rI∗−sR∗.

After some manipulations, we obtain the following solutions:

(14) I∗=−αNd,R∗=αNsd,S∗=d+ri.

According to the survey done in the last months, it was revealed that the disease has a high rate of mortality with a maximum of 90 percent [12]. This implies the recovery rate is very small, and then the existence conditions are true and are conformable to the real world situation.

### 3.3. Eigen-Values Solutions

We shall make use of the Jacobian method to find the Eigen-value associate of the endemic equilibrium points. The Jacobian matrices associate is given as

(15) $$\begin{array}{c} J=\left(\begin{array}{ccc} -iI & -iS & s\\ -iI & -i-d-r & 0\\ 0 & r & -s \end{array}\right). \end{array}$$

However, the free disease equilibrium point where (((*d* + *r*)/*i*), 0, 0), we have the following:

(16) $$\begin{array}{c} J=\left(\begin{array}{ccc} 0 & -\left(d+r\right) & s\\ 0 & -i-d-r & 0\\ 0 & r & -s \end{array}\right). \end{array}$$

To find the Eigen-value associate, we solve the following equation:

(17) aaaaaaaaaadet⁡J−Iλ=0,det⁡−λ−d+rs0−i−d−r−λ00r−s−λ=0.

And the solutions of the above equation are given as

(18) λ=0,λ=−2r+d,λ=−s.

## 4. Derivation of the Solution

We shall in this section present the derivative of the solution for system (2). Since the system is nonlinear, some analytical techniques such as Laplace transform, Fourier transform, and Green function will not be suitable for this case. Suitable methods for nonlinear equations have been documented in the literature, for instance, homotopy perturbation method and its derivatives [13–15] and variational iteration method and its derivatives [16–18]. However, in this work, we shall use the homotopy decomposition method [19, 20].

In this method, we first apply the inverse operator of _0_^*A*^ *D*_*t*_^*β*^ defined in (8) on system (5) to obtain

(19) St=S0+I0Atβ−iStIt+sRt−αN,It=I0+I0AtβiStIt−dIt−rIt,Rt=R0+I0AtβrIt−sRt,Dt=D0+I0AtβdIt+αN.

The next move in this method is to assume that, since the system is nonlinear, then the solutions can be obtained in series as

(20) St=lim⁡p→1⁡∑n=0∞pnSnt,It=lim⁡p→1⁡∑n=0∞pnInt,Rt=lim⁡p→1⁡∑n=0∞pnRnt,Dt=lim⁡p→1⁡∑n=0∞pnDnt,aaaaaaaaaaaa1<p≤1.

However, introducing the above proposed solution into (19), also making use of the idea of homotopy and after we compare terms of the same power of *p*, we obtain the following:

(21) p0:S0t=S0I0t=I0R0t=R0D0t=D0,p1:S1t =I0Atβ−iS0tI0t+sR0t−αNI1t =I0AtβiS0tI0t−dI0t−rI0tR1t=I0AtβrI0t−sR0tD1t=I0AtβdI0t+αN,p2:S2t =I0Atβ−iS1tI0t−iS0tI1t+sR1tI2t =I0Atβ−iS1tI0t−iS0tI1tMMM −dI1t−rI1tR2t=I0AtβrI1t−sR1tD2t=I0AtβdI1t.

For any *n* ≥ 2, we have the following:

(22) pn:Snt =I0Atβ−∑j=0n−1iSjtIn−j−1t+sRntInt=I0Atβ∑j=0n−1iSjtIn−j−1t−dIn−1t−rIn−1tRnt=I0AtβrIn−1t−sRn−1tDnt=I0AtβdIn−1t.

One of the important parts of any iteration method is to prove the uniqueness and the convergence of the method; we are going to show the analysis underpinning the convergence and the uniqueness of the proposed method for the general solution for *p* = 1.

**Theorem 3 .**

Assuming that *X* and *Y* are Banach spaces and *V* : *X* → *Y* is contraction nonlinear mapping. If the progression engender by the three-dimensional homotopy decomposition method is regarded as

(23) $$\begin{array}{c} S_{n}\left(t\right)=V\left(S_{n-1}\left(t\right)\right)=\overset{n-1}{\underset{k=0}{\sum }}S_{k}\left(t\right),\;n=1,2,3\ldots , \end{array}$$

then, the following statements hold:

- ||*S*_*n*_(*t*) − *S*(*t*)|| ≤ *ρ* ^*n*^||*S*(0) − *S*(*t*)||,* with*  0 < *ρ* < 1;
- For any other *n* greater than 0, *S*_*n*_(*t*) is always in the neighborhood of the exact solution *S*(*t*);
- lim⁡_*n*→*∞*_ *S*_*n*_(*t*) = ⁡*S*(*t*).

**Proof**

The proof of (a) shall be achieved via induction on the natural number *n*. When *n* = 1, we have the following:

(24) $$\begin{array}{c} ||S_{1}\left(t\right)-S\left(t\right)||=||V\left(S_{0}\left(t\right)\right)-S\left(t\right)||. \end{array}$$

However, by hypothesis, we have that *V* has a fixed point, which is the exact solution. Because

if *S*(*t*) is the exact solution, then,

(25) StS∞x,y,t=V∑k=0∞−1Skt=V∑k=0∞Skt=∑k=0∞Skt;

since *∞* − 1 is the same as *∞*, therefore, we have that

(26) $$\begin{array}{c} S\left(t\right)=V\left(S\left(t\right)\right). \end{array}$$

Then,

(27) $$\begin{array}{c} ||S_{1}\left(t\right)-S\left(t\right)||=||V\left(S_{0}\left(t\right)\right)-V\left(S\left(t\right)\right)||. \end{array}$$

Since *V* is a contractive nonlinear mapping, we shall have the following inequality:

(28) VS0t−VSt≤ρS0t−St, 0<ρ<1;

then, the property is verified for *n* = 1. Assume that the hypothesis is verified for *n* − 1; we shall prove that it is also verified for *n*. However, at the level *n*, we have the following:

(29) Snt−StVSn−1t−St=VSn−1t−VSt.

Using the fact that *V* is a nonlinear contractive mapping, we have the following:

(30) $$\begin{array}{c} ||V\left(S_{n-1}\left(t\right)\right)-V\left(S\left(t\right)\right)||<\rho ||S_{n-1}\left(t\right)-V\left(S\left(t\right)\right)||. \end{array}$$

Furthermore, using the induction hypothesis, we arrive at

(31) $$\begin{array}{c} \rho ||S_{n-1}\left(t\right)-V\left(S\left(t\right)\right)||<\rho \rho^{n-1}||S_{0}\left(t\right)-S\left(t\right)||. \end{array}$$

And the proof is completed. The proof of (b) and (c) can be found [19].

**Figure 3:**
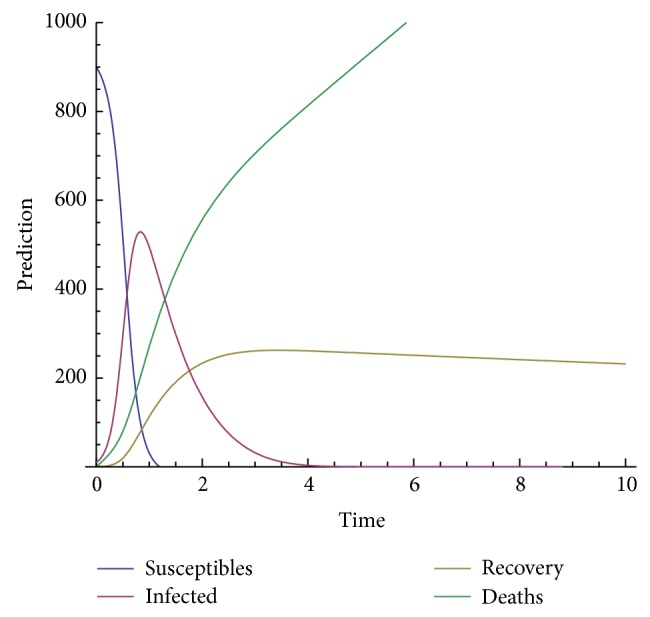
Prediction for beta = 1.

**Figure 4:**
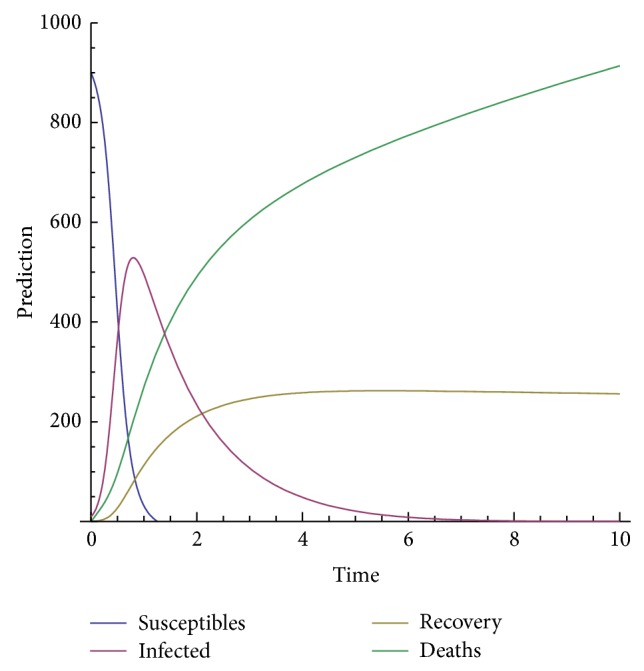
Prediction for beta = 0.5.

**Figure 5:**
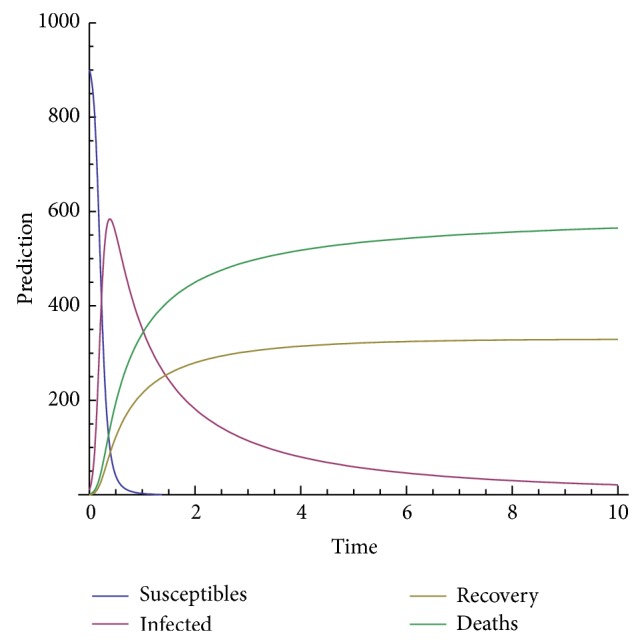
Prediction for beta = 0.2.

**Figure 6:**
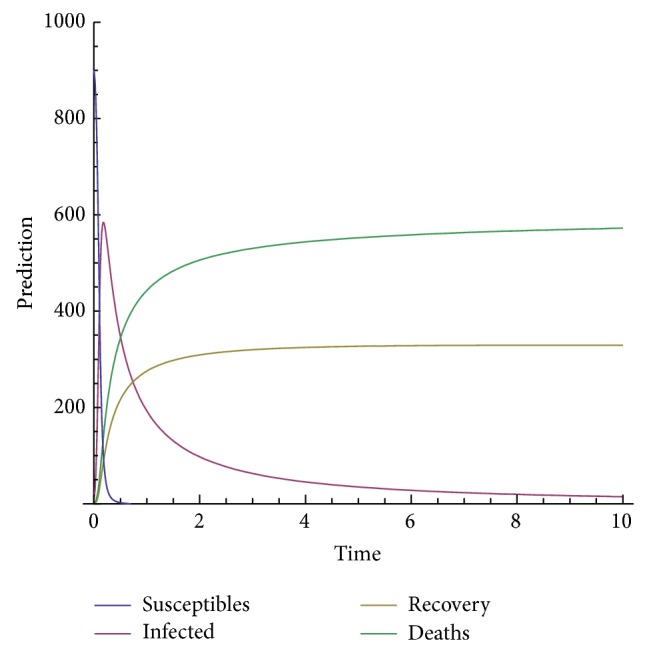
Prediction for beta = 0.1.

**Figure 7:**
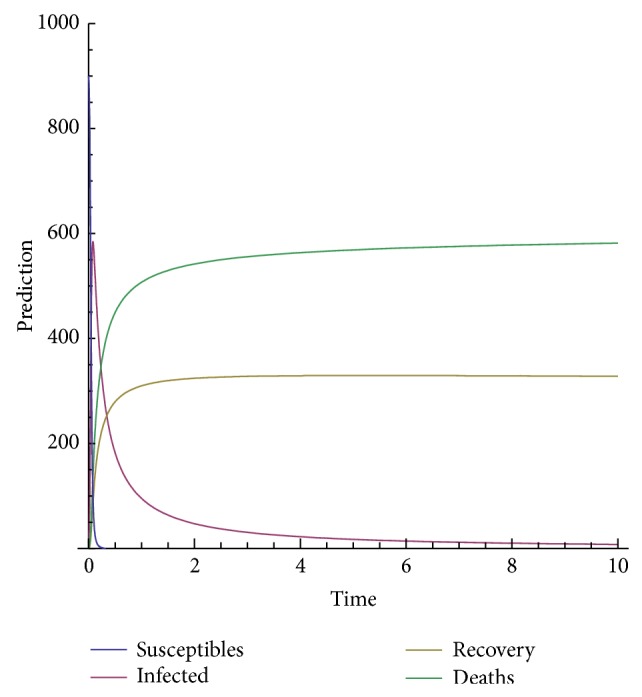
Prediction for beta = 0.05.

In order to show the efficiency and applicability of this method for handling the system nonlinear equations we shall present some numerical solution in Figures 3, 4, 5, 6, and 7.

The model depends on the order of the derivative; according to the prediction, when beta is 1, meaning the model with the classical derivative, the total number of individuals in the given West African country will all die, which is not realistic because we assume that, amount the 1000 persons living in a given West African country, only 900 are susceptible. We are expecting that, in worse case, all the 900 susceptible will die and only those who are not susceptible will live. Now, we observe from the figure that, for beta less than 0.5, the model predicts that, at the time *t* = 10, about 540 will be dead, about 300 will recover from the disease, about 40 will be infected, and only about 10 will be susceptible.

## 5. Conclusion

The derivative used to model a real world situation is very important. The classical derivative describes the change of rate, but it is an approximation of the real velocity of the object under study; this study has been examined in detail in [6]. The beta derivative is the modification of the classical derivative that takes into account the time scale and also has new parameter that can be considered as the fractional order. We have used the beta derivative to model the spread of the fatal disease called Ebola that has killed many people in the West African countries including Nigeria, Sierra Leone, Guinea, and Liberia since December 2013. We did the investigation of the stable endemic points and presented the Eigen-values using the Jacobian method. The homotopy decomposition method was used to solve the resulted system of equations. The convergence of the method was presented and some numerical simulations were done for different values of beta. The simulations showed that our model is more realistic for all betas less than 0.5. The model revealed that, for a given population in a West African country, if there were no precaution of recovering, even if the total number of infected populations is very small, the entire population of that country would all die in a very short period of time. Based on the prediction of this paper, we are calling upon more research around this disease; in particular, we are calling for researchers to pay their attention to finding a very good cure or a better prevention, to reduce the risk of contamination.

## Figures and Tables

**Table 1:** Parameters used for simulations according to some reported data.

Parameters	Values
*S*(0)	900
*I*(0)	10
*R*(0)	0
*D*(0)	0
*N*	1000
*α*	0.01
*r*	0.4
*i*	0.01
*s*	0.02
*d*	0.6
